# Outcomes in Establishing Individual Vessel Patency for Pediatric Pulmonary Vein Stenosis

**DOI:** 10.3390/children8030210

**Published:** 2021-03-10

**Authors:** Ryan Callahan, Kimberlee Gauvreau, Audrey C. Marshall, Laureen M. Sena, Christopher W. Baird, Christina M. Ireland, Kerry McEnaney, Elsa C. Bjornlund, Juliana T. Mendonca, Kathy J. Jenkins

**Affiliations:** 1 Department of Cardiology, Boston Children’s Hospital and Harvard Medical School, Boston, MA 02115, USA; Kimberlee.Gauvreau@CARDIO.CHBOSTON.ORG (K.G.); audrey.marshall@sickkids.ca (A.C.M.); Christina.Ireland@CARDIO.CHBOSTON.ORG (C.M.I.); Kerry.McEnaney@childrens.harvard.edu (K.M.); Elsa.Bjornlund@cardio.chboston.org (E.C.B.); Juliana.Mendonca@childrens.harvard.edu (J.T.M.); kathy.jenkins@cardio.chboston.org (K.J.J.); 2 Department of Radiology, Boston Children’s Hospital and Harvard Medical School, Boston, MA 02115, USA; Laureen.Sena@childrens.harvard.edu; 3 Department of Cardiac Surgery, Boston Children’s Hospital and Harvard Medical School, Boston, MA 02115, USA; Chris.Baird@cardio.chboston.org

**Keywords:** congenital heart disease, drug therapy, treatment, outcome

## Abstract

The purpose of this study was to determine what patient and pulmonary vein characteristics at the diagnosis of intraluminal pulmonary vein stenosis (PVS) are predictive of individual vein outcomes. A retrospective, single-center, cohort sub-analysis of individual pulmonary veins of patients enrolled in the clinical trial NCT00891527 using imatinib mesylate +/− bevacizumab as adjunct therapy for the treatment of multi-vessel pediatric PVS between March 2009 and December 2014 was performed. The 72-week outcomes of the individual veins are reported. Among the 48 enrolled patients, 46 patients and 182 pulmonary veins were included in the study. Multivariable analysis demonstrated that patients with veins without distal disease at baseline (odds ratio, OR 3.69, 95% confidence interval, CI [1.52, 8.94], *p* = 0.004), location other than left upper vein (OR 2.58, 95% CI [1.07, 6.19], *p* = 0.034), or veins in patients ≥ 1 y/o (OR 5.59, 95% CI [1.81, 17.3], *p* = 0.003) were at higher odds of having minimal disease at the end of the study. Veins in patients who received a higher percentage of eligible drug doses required fewer reinterventions (IRR 0.76, 95% CI [0.68, 0.85], *p* < 0.001). The success of a multi-modal treatment approach to aggressive PVS depends on the vein location, disease severity, and drug dose intensity.

## 1. Introduction

Pediatric intraluminal pulmonary vein stenosis (PVS) is caused by the hyperplasia of myofibroblast-like cells in a myxocollagenous matrix which compromises flow and leads to pulmonary hypertension, right heart failure, and death [1,2,3]. Factors associated with patient survival include age, overall disease burden, the severity of pulmonary hypertension, and right ventricular function [4,5,6,7,8,9]. Aggressive subtypes involve multiple veins and have a high incidence of restenosis, irrespective of intervention [10,11,12]. In these cases, a multi-modal approach using transcatheter and surgical techniques combined with adjunct anti-proliferative therapy is implemented in an effort to improve survival [13]. While patient outcomes from various single center and multi-center studies have been well reported, investigations on the outcomes of the individual pulmonary veins are limited. Given that each individual pulmonary vein (right upper, right lower, left upper, left lower) has its own geometric shape and unique surrounding anatomy, it may be advantageous to explore how pulmonary vein location affects their outcome. Further, as patient survival is dependent on the preservation of as much healthy pulmonary vein bed as possible, identifying individual pulmonary vein characteristics associated with vein outcomes may assist in improving patient survival. For this study, we hypothesized that pulmonary vein location and pulmonary vein disease severity at baseline would predict vein outcome.

## 2. Materials and Methods

### 2.1. Study Aims

The purpose of this study was to determine what patient and pulmonary vein characteristics at initial evaluation are predictive of individual pulmonary vein outcomes. The primary outcome is the status (disease severity) of the individual veins at 72 weeks, or prior to study exit secondary to death or lung transplant (study endpoint). The secondary outcome is the number of reinterventions (surgery or transcatheter) for each vein during the study period.

### 2.2. Study Design

This was a retrospective, single-center, cohort sub-analysis of patients enrolled in the single-arm, prospective, open-label, United States Food and Drug Administration approved clinical trial (NCT00891527) at Boston Children’s Hospital (BCH) using biologic inhibition agents imatinib mesylate (Gleevec^®^, Novartis Inc., Basel, Switzerland) and bevacizumab (Avastin^®^, Genentech Inc., South San Francisco, CA, USA) as adjunct therapy for the treatment of multi-vessel PVS [13]. Please refer to previous publications for the full study protocol and a description of the surgical technique [11,13]. In brief, 48 patients with at least 2 vessel PVS (median: 4 (range 2–5)) with a mean gradient of ≥4 mmHg by echocardiography or catheterization were enrolled between March 2009 and December 2014. Patients underwent surgical or transcatheter intervention for the relief of PVS, followed by at least 48 weeks of drug therapy paired with transcatheter/surgical reinterventions, if necessary, for pulmonary vein restenosis. Most patients underwent surgical repair prior to drug initiation, as this was the preferred approach. Those patients who had a transcatheter intervention prior to drug initiation had a history of previous pulmonary vein surgical repair or had a contraindication to surgery at the time of study enrollment. Drug therapy (imatinib mesylate 340 mg/m^2^ per oral once daily plus/minus bevacizumab 10 mg/kg intravenous every 2 weeks) was initiated 7 to 10 days after surgery in order to allow for adequate wound healing (initiated post-operative day 1 if transcatheter intervention only). Patients with congenital heart disease received imatinib mesylate only and bevacizumab was added later if PVS progression occurred in order to mitigate the potential risk of central line access. Patient indications for reintervention included changes in clinical status (changes in respiratory status, feeding intolerance), echocardiogram (increasing gradients, right ventricle [RV] hypertension, RV dysfunction), and/or nuclear lung perfusion scan. When to perform a transcatheter reintervention on a pulmonary vein was at the discretion of the operator, but in general required at least mild ostial stenosis. Pulmonary vein status was categorized (Table 1) using computed tomography (CT) or cardiac angiography (gold standard) at baseline (following initial surgical/transcatheter intervention and drug initiation), 24 weeks, 48 weeks, and 72 weeks using pre-established criteria assigned by the BCH core lab readers (R.C., A.C.M., L.M.S.). The efficacy and safety outcomes were assessed at 48 and 72 weeks. For the purpose of this study, a new baseline (referred to as “baseline” moving forward) pulmonary vein status was established using CT or cardiac angiography (RC) prior to the initial intervention and drug initiation. Vein categories were further condensed into no disease (category 1), proximal disease (category 2–4), distal disease (category 5–6), and distal atresia (category 7). The four main lobar pulmonary veins were analyzed (right upper, right lower, left upper, left lower) for this study, with the exclusion of the right middle and lingula pulmonary veins. The study endpoint was at 72 weeks or prior to study exit, such as death or lung transplant, in which case the most recent preceding image was used for vein categorization. Patients with no imaging data following study enrollment due to death were excluded.

### 2.3. Outcomes and Data Collection

For the primary outcome, the status of the individual pulmonary veins at the study endpoint was dichotomized as having minimal disease (category 1 or 2) or more than minimal disease (category 3–7). The secondary outcome was the rate of transcatheter or surgical reinterventions on the individual pulmonary veins during the study period, following initial surgical/transcatheter intervention and drug initiation. Predictor variables include the vein location, vein status at baseline, the percentage of eligible drug doses received by the patient during the study period, and whether the pulmonary vein was part of a common vein at baseline prior to surgical intervention. A common vein was defined as the joining of the upper and lower pulmonary veins prior to entering the left atrium. Potential confounders include age at the start of drug therapy, gender, underlying diagnosis, single ventricle physiology, the presence of lung disease (bronchopulmonary dysplasia, lung hypoplasia, airway malacia, interstitial lung disease, congenital lobar emphysema), history of prematurity (gestation < 37 weeks), concomitant genetic syndrome, and the type of initial intervention prior to start of drug therapy. Data were collected from the BCH clinical trial core lab, the institutional PVS registry, and patient medical records.

### 2.4. Statistical Analysis

Baseline patient and vessel characteristics are described using frequencies and percentages for categorical variables and medians, ranges, and interquartile ranges for continuous variables. Logistic regression was used to assess the relationships between patient and vein characteristics at baseline and the primary outcome variable at the study endpoint. Poisson regression was used to assess the relationships between the patient and vein characteristics and the rate of reinterventions on each vein. For both the logistic and Poisson models, standard errors of the regression coefficients were estimated accounting for the lack of independence of multiple veins within the same patient. All the variables with *p* < 0.20 by univariate analysis were considered for inclusion in multivariable models; *p* < 0.05 by the likelihood ratio test was required for retention in the final model. Veins with baseline distal atresia were excluded from the analysis because there was no attempt to recannalize by surgical or transcatheter intervention.

### 2.5. Ethics Statement

All the subjects gave their informed consent for inclusion before they participated in the study. The study was conducted in accordance with the Declaration of Helsinki, and the protocol was approved by the Boston Children’s Hospital Institutional Review Board (protocol number: 07-06-0249, date of approval: 6 September 2008).

## 3. Results

### 3.1. Patients

Of the 48 reported patients from the trial, 2 died prior to obtaining follow-up images, and thus 46 patients and 182 pulmonary veins (4 veins per patient, 1 patient born with only 1 lung/2 veins) were included in the study (Table 2). Most patients were less than one year of age (36/46; 78%), had four vessel involvement at baseline (25/46; 54%), and had PVS associated with congenital heart disease (37/46; 80%, 16 of which had anomalous pulmonary venous connections).

### 3.2. Vein Status

**Figure 1:**
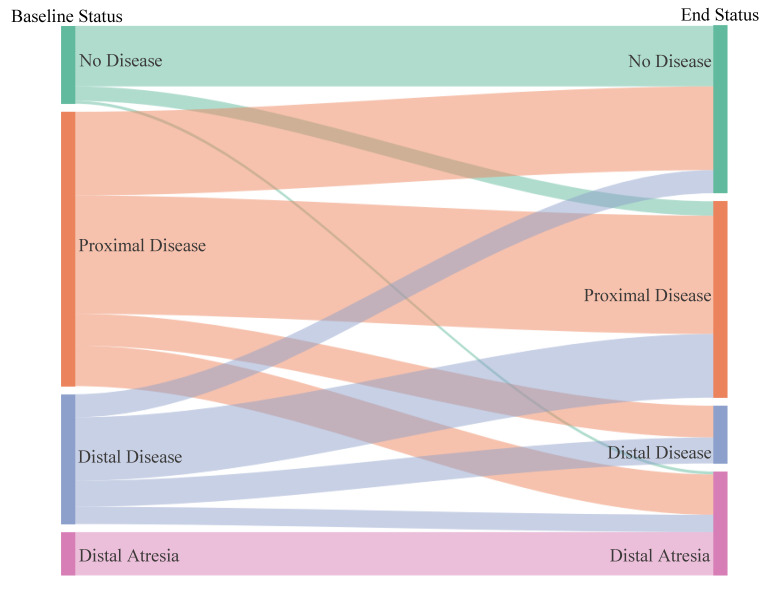
Change in disease status of all pulmonary veins, baseline to study endpoint.

**Figure 2:**
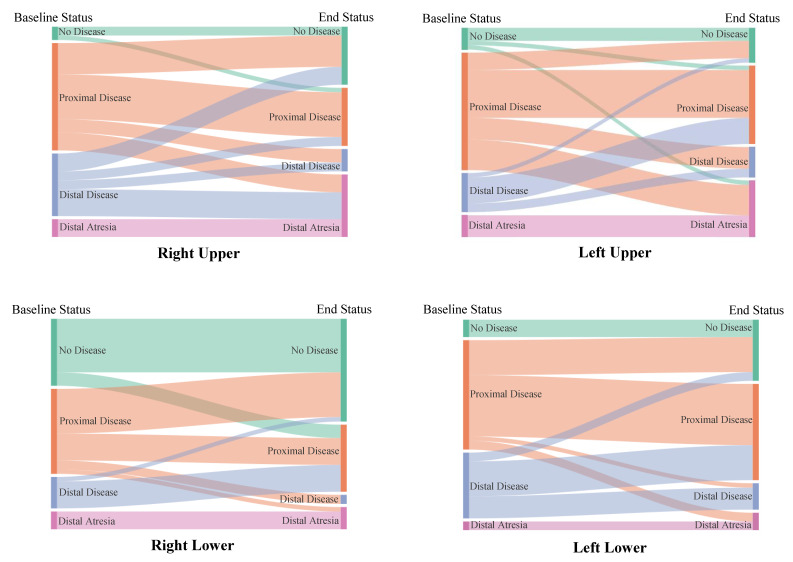
Change in disease status by pulmonary vein location, baseline to study endpoint.

At baseline, of the 182 pulmonary veins 27 (15%) had no disease, 95 (52%) had proximal disease, 45 (25%) had distal disease, and 15 (8%) had distal atresia (Table 3). Twenty-eight veins (15%) were part of a common vein. At the study endpoint, of the 182 pulmonary veins 58 (32%) had no disease, 68 (37%) had proximal disease, 20 (11%) had distal disease, and 36 (20%) had distal atresia (Figure 1 and Figure 2). Of the 27 veins with no disease at baseline, 6 developed disease during the study period.

### 3.3. Primary Outcome

**Figure 3:**
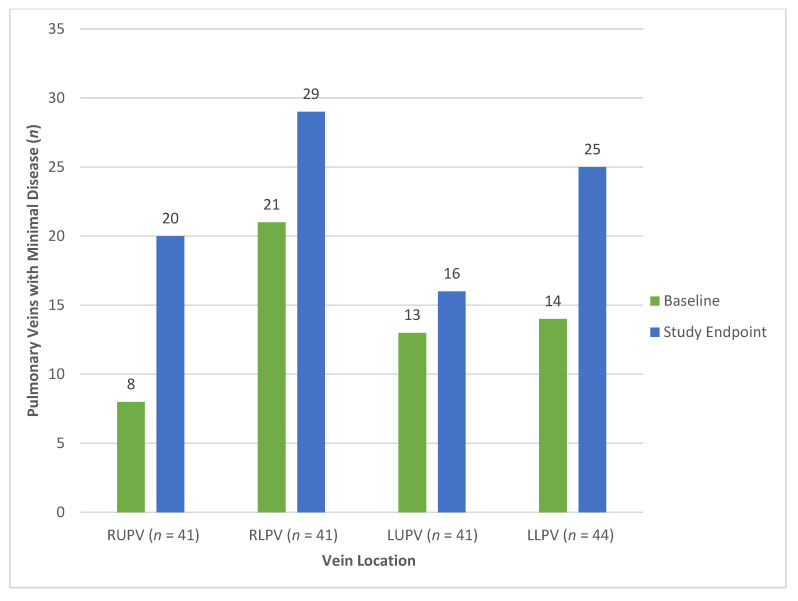
Number of individual pulmonary veins with minimal disease at baseline and the study endpoint. RUPV = right upper pulmonary vein, RLPV = right lower pulmonary vein, LUPV = left upper pulmonary vein, LLPV = left lower pulmonary vein.

At the study endpoint, 90/182 (50%) of the pulmonary veins had minimal disease, while 77/182 (42%) had more than minimal disease; the remaining 15/182 (8%) veins with distal atresia at baseline were excluded from analysis. Figure 3 illustrates the number of individual pulmonary veins with minimal disease at baseline and the study endpoint. In a univariate analysis (Table 4), veins in patients ≥ 1 year of age at drug initiation (*p* = 0.001), veins with no disease at baseline (*p* = 0.001), veins with no distal disease at baseline (*p* = 0.001), and right lower vein location (*p* = 0.006) were more likely to have minimal disease at the study endpoint. On the contrary, the left upper vein (*p* = 0.013) and veins with primary PVS (*p* = 0.051) were less likely to have minimal disease at the study endpoint. In a multivariable analysis (Table 5), veins in patients ≥ 1 year of age at drug initiation (odds ratio, OR 5.59, 95% confidence interval, CI [1.81, 17.3], *p* = 0.003), veins with no disease at baseline (OR 6.15, 95% CI [1.69, 22.4], *p* = 0.006), veins with no distal disease at baseline (OR 3.69, 95% CI [1.52, 8.94], *p* = 0.004), and vein location not the left upper (OR 2.58, 95% CI [1.07, 6.19], *p* = 0.034) were more likely to have minimal disease.

### 3.4. Secondary Outcome

During the study period (following initial surgical/transcatheter intervention and drug initiation), excluding the 15 veins with distal atresia, 85/167 (51%) veins underwent no reinterventions, while 25/167 (15%) underwent 1 reintervention and 57/167 (34%) underwent ≥ 2 reinterventions. In the univariate analysis (Table 6), veins in patients ≥ 6 months of age at drug initiation (*p* = 0.011), veins in patients who received more of their eligible drug doses (*p* < 0.001), veins with no disease at baseline (*p* = 0.036), and veins that were part of a common vein (*p* = 0.007) were less likely to require reinterventions. Veins with distal disease at baseline (*p* = 0.022) were more likely to require reinterventions. In the multivariable analysis (Table 7), veins in patients who received more of their eligible drug doses (incidence rate ratio, IRR 0.76, 95% CI [0.68, 0.85], *p* < 0.001), veins with no disease at baseline (IRR 0.18, 95% CI [0.05, 0.67], *p* = 0.011), and veins that were part of a common vein (IRR 0.17, 95% CI [0.05, 0.55], *p* = 0.003) were less likely to require reinterventions. 

## 4. Discussion

Intraluminal pulmonary vein stenosis is a complex and vexing disease that typically occurs in a young and hemodynamically fragile patient population. PVS is unified by its cellular composition and its secondary insults to the lungs and pulmonary artery vasculature, but the patients as a whole are quite heterogeneous and challenging to study [3,14]. Intuitively, the higher the number of pulmonary veins involved in a patient and the more severe disease that is present within each vein, the worse the patient outcome [4,5,6,7,9,15]. Efforts to treat PVS either mechanically or pharmacologically and their measured success have historically been applied to the whole patient and not to the individual pulmonary veins. What is not known is whether each individual pulmonary vein, with its own unique geometric shape and surrounding anatomy, has characteristics that lead to the success or failure of specific therapies. Thus, this study was an effort to broaden our understanding of what basic individual vein characteristics are associated with pulmonary vein outcomes and need for reinterventions. Our study identified that patient age, vein location, and disease severity at baseline were all associated with vein outcome. Specifically, veins in older patients, veins with no distal disease at baseline, and veins in a location other than the left upper did better. Furthermore, we determined that veins with no disease at baseline, veins which were part of a common vein, and veins in patients who received a higher drug (imatinib) intensity were less likely to require reinterventions during the study period.

While, to the best of our knowledge, there are no studies on individual pulmonary vein outcomes to compare our work to, there were similarities between the vein outcomes and previously published patient outcomes. First, patients who are diagnosed with PVS at less than six months of age are at a higher risk of mortality [4,5,9]. This speaks to the aggressive nature of the disease in infants, particularly the primary subtype (ex-full term, structurally normal heart), the ongoing stimulus for restenosis such as lung disease, and the challenges to intervention in small patients [1,11,13]. Furthermore, given the small baseline size of pulmonary veins in infants as compared to older children, less neo-intimal hyperplasia may be required in order to create a critical stenosis. Second, patients with pulmonary veins that have evidence of distal or upstream disease by cardiac angiography, computed tomography, or cardiac magnetic resonance also tend to do poorly [6,7,15]. The distal luminal narrowing occurs as a result of PVS progression from diffuse fibromyxoid proliferation, venous arterialization, and/or flow redistribution to unaffected or less affected lung segments [14,16,17]. The early diagnosis and treatment of proximal PVS appears to be crucial in order to optimize vein and patient outcomes. However, it is noteworthy that in our study most veins with distal disease without atresia were salvaged and had either no disease or proximal disease at the study endpoint (Figure 1). Thus, treated veins can undergo positive remodeling and/or restore flow (and distal caliber) once the proximal obstruction is relieved. Lastly, the patients in the clinical trial used for this study who received a higher percentage of eligible drug (imatinib) doses—i.e., drug intensity—were more likely to stabilize, which was defined as no reinterventions for 6 months (13). Our by-vessel analysis of the individual veins confirmed this finding. Chemotherapy dose intensity has been shown to correlate with outcomes and this includes imatinib in the treatment of chronic myeloid leukemia [18,19]. We aim to frequently optimize imatinib dose for changes in body surface area in our PVS patients, encourage compliance, and hold the medication only when necessary (most commonly for fever evaluation or surgery).

Vein location influenced the vein outcome in our study. Specifically, the left upper pulmonary veins were less likely to have minimal disease at the study endpoint. In univariate analysis, the right lower pulmonary veins had higher odds of having minimal disease. Our group and others have theorized that PVS occurs as the result of disturbed flow and changes to vein wall shear stress in susceptible patients [6,20,21]. Each individual vein is surrounded by unique anatomy that can potentially distort or compress the vein and act as a nidus for PVS—i.e., the left upper and left mainstem bronchus, the right upper and right pulmonary artery, and the left lower and descending aorta and heart mass. The pericardial reflection may also play a role in fixing the pulmonary veins, allowing for further vein distortion. These mechanisms can become exacerbated as the left bronchus dilates from chronic ventilation, the right pulmonary artery dilates from secondary pulmonary hypertension, and the heart rotates and dilates from chronic left lower lobe atelectasis and left to right shunts, respectively. The right lower, with no adjacent structures and a short intra-pericardial distance from the pericardial reflection to the entrance into the left atrium, is usually free of distortion/compression, although there are exceptions (heart rotation from left lower lobe atelectasis and/or cardiomegaly causing a proximal kink). Thus, with no nidus for turbulence, it was not surprising to see the right lower vein be the vein to most likely have no disease at baseline and ultimately have a better outcome (Figure 2 and Figure 3). The multiple areas of distortion (relationship to pericardial reflection, anterior distortion from left mainstem bronchus followed by posterior entry into the left atrium) and the fact that it is the smallest vein draining the least amount of lung territory could potentially explain why the left upper was more likely to have a poor outcome.

Pulmonary veins with no disease at baseline were more likely to have minimal disease at the study endpoint and were less likely to require reinterventions during the study period. Six of the vessels developed PVS during the study period, hypothetically due to the redistribution of blood flow from affected lobes, leading to increased disturbed flow and neo-intimal proliferation. This stresses the importance of active surveillance in patients with PVS—monitoring for restenosis in previously affected veins and new stenosis in previously unaffected veins. It is unclear if the targeted anti-proliferative therapy, which aims to stop or slow neo-intimal proliferation, prevented new disease in the unaffected veins.

Pulmonary veins that were initially part of a common vein were less likely to require reintervention during the study period. All the common veins underwent a sutureless-type repair prior to drug initiation, resolving the proximal stenosis. This suggests that the turbulent funneling of blood flow into a common vein may play a role in the mechanism of PVS. Removing this turbulence by surgically expanding the connection of the left atrium to the individual veins concurrently with targeted anti-proliferative therapy may reduce the incidence of restenosis and the need for reintervention.

With the high incidence of restenosis following surgical pulmonary vein repair with sutureless techniques (no difference in vein outcomes between conventional and modified sutureless in our study) and the understanding that disturbed vein flow could play a role in PVS, we have evolved our institutional practice [11]. We approach each vein individually and perform an anatomic repair with the aim to identify the mechanism of obstruction and turbulence followed by changing the geometry of the vein in an effort to promote laminar flow. In the case of left upper pulmonary vein stenosis, for example, the pericardial reflection is mobilized and the vein is brought anteriorly away from the left mainstem bronchus and then re-implanted/patched to the base of the left atrial appendage. The results of this anatomic focused repair strategy are currently under investigation [22].

### Limitations

The categorization of the pulmonary vein disease severity was strictly based on the luminal appearance from imaging data. Other important hemodynamic and physiology outcome variables such as pulmonary vein gradients and quantified lobar perfusion were not considered in this study. This study assumed an adequate correlation between CT and angiography for pulmonary vein status categorization [23]. The type of reintervention performed during the study period and its effect on the outcomes was not assessed in this study. The role of dual therapy (imatinib mesylate and bevacizumab) was not analyzed, as bevacizumab was given to patients with more aggressive PVS subtypes or patients who failed imatinib mesylate introducing bias. While the management of PVS has evolved since that the last patient was enrolled in the trial (2014), its learning points are relevant today and can serve as a foundation for future investigations with the goal of developing an individual pulmonary vein treatment strategy. As a result of this study, more specific individual vein characteristics and how they relate to vein outcomes will be investigated in an upcoming prospective PVS study.

## 5. Conclusions

In pediatric patients with aggressive, multi-vessel intraluminal pulmonary vein stenosis, patient and pulmonary vein characteristics influence the outcomes of the individual pulmonary veins. The success of a multi-modal treatment approach (transcatheter/surgical interventions, targeted anti-proliferative therapy) in the treatment of individual pulmonary vein stenosis depends on patient age, pulmonary vein location, the extent of vein disease at diagnosis, and drug dose intensity.

## Figures and Tables

**Table 1:** Pulmonary vein status categorical criteria.

Category	Criteria
1	None: no narrowing of the luminal contour.
2	Mild proximal narrowing: mild (<50%) narrowing of the proximal (<5 mm) luminal contour.
3	Proximal atretic: complete obliteration of the luminal contour confined to the proximal (<5 mm) vessel segment (distal vasculature unaffected).
4	Significant proximal narrowing: significant (>50%) narrowing of the proximal (<5 mm) luminal contour.
5	Extensive narrowing: narrowing of the luminal contour confined to a defined portion of the vessel but extending > 5 mm beyond the left atrium.
6	Diffuse: narrowing of the entire luminal contour.
7	Distal atretic: complete obliteration of the luminal contour extending >5 mm within the vessel segment.

**Table 2:** Patient characteristics.

Patient Characteristics (*n* = 46)	*N* (%) or Median [Interquartile Range] (Range)
Age at drug start date (months)	7 [5, 12] (1, 61)
Age at drug start date	
→ <6 months	17 (37%)
→ 6–11 months	19 (41%)
→ ≥1 year	10 (22%)
Sex female	21 (46%)
Premature birth (<37 weeks gestation)	18 (39%)
If premature, gestational age (week)	32 [29, 35] (25, 36)
Diagnosis	
→ CHD	21 (46%)
→ CHD/TAPVC	13 (28%)
→ CHD/PAPVC	3 (7%)
→ Primary PVS	4 (9%)
→ Isolated lung disease	5 (11%)
Single ventricle physiology	6 (13%)
Presence of lung disease	17 (37%)
Genetic syndrome	13 (28%)
Number of veins affected at baseline	
→ 2	8 (17%)
→ 3	13 (28%)
→ 4	25 (54%)
Percent of eligible drug doses received (*n* = 44)	0.86 [0.75, 0.92] (0.54, 0.99)
Surgery type prior to drug start	
→ None	4 (9%)
→ Conventional sutureless	19 (41%)
→ Modified sutureless	19 (41%)
→ Other ^1^	4 (9%)

^1^ Other surgeries include ostial resection and reimplantation and the debridement/enlargement of orifice. CHD = congenital heart disease, TAPVC = total anomalous pulmonary venous connection, PVS = pulmonary vein stenosis.

**Table 3:** Change in disease status, baseline to study endpoint.

			PVS Status at Study Endpoint						
PVS Status at Baseline		*n*	1	2	3	4	5	6	7
1	No disease	27	21 (78)	3 (11)	0 (0)	2 (7)	0 (0)	0 (0)	1 (4)
2	Mild proximal narrowing	29	11 (38)	7 (24)	0 (0)	5 (17)	3 (10)	1 (3)	2 (7)
3	Proximal atretic	4	0 (0)	1 (25)	0 (0)	1 (25)	0 (0)	1 (25)	1 (25)
4	Significant proximal narrowing	62	18 (29)	16 (26)	0 (0)	11 (18)	5 (8)	1 (2)	11 (18)
5	Extensive narrowing	30	8 (27)	3 (10)	1 (3)	9 (30)	2 (7)	4 (13)	3 (10)
6	Diffuse	15	0 (0)	2 (13)	3 (20)	4 (27)	0 (0)	3 (20)	3 (20)
7	Distal atretic	15	0 (0)	0 (0)	0 (0)	0 (0)	0 (0)	0 (0)	15 (100)
	Total	182	58	32	4	32	10	10	36

Value is shown as number (%). PVS = pulmonary vein stenosis.

**Table 4:** Vein status at study endpoint, by patient, and vessel characteristics: univariate analysis for the outcome of minimal disease.

	Minimal Disease ^1^	More Than Minimal Disease ^1^	Odds Ratio	95% Confidence Interval	*p* Value
	(*n* = 90)	(*n* = 77)			
Patient Level					
Age at drug start date (months)	9 (1, 61)	6 (1, 45)	1.07	(1.01, 1.12)	0.017
Age at drug start date					
→ <6 months	24 (27%)	37 (48%)	1.00	-	-
→ 6 months to <1 year	39 (43%)	32 (42%)	1.88	(0.78, 4.51)	0.16
→ ≥1 year	27 (30%)	8 (10%)	5.20	(2.00, 13.5)	0.001
Sex female	45 (50%)	32 (42%)	1.41	(0.63, 3.16)	0.41
Premature birth	39 (43%)	28 (36%)	1.34	(0.62, 2.90)	0.46
Diagnosis primary PVS	4 (4%)	12 (16%)	0.25	(0.06, 1.00)	0.051
Diagnosis anomalous vein	27 (30%)	24 (31%)	0.95	(0.42, 2.11)	0.89
Single ventricle physiology	13 (14%)	10 (13%)	1.13	(0.38, 3.34)	0.82
Presence of lung disease	38 (42%)	21 (27%)	1.95	(0.81, 4.69)	0.14
Percent of eligible drug doses received (↑5%) (*n* = 86, 73)	0.87 (0.81, 0.93)	0.85 (0.72, 0.90)	1.08	(0.94, 1.26)	0.27
Surgery type					
→ Conventional sutureless	37 (41%)	35 (45%)	1.00	-	-
→ Modified sutureless	36 (40%)	33 (43%)	1.03	(0.44, 2.41)	0.94
→ None/Other	17 (19%)	9 (12%)	1.79	(0.54, 5.91)	0.34
Vein Level					
No distal disease at baseline	77 (86%)	45 (58%)	4.21	(1.86, 9.54)	0.001
No disease at baseline	24 (27%)	3 (4%)	8.97	(2.43, 33.0)	0.001
Location right upper	20 (22%)	21 (27%)	0.76	(0.41, 1.41)	0.39
Location right lower	29 (32%)	12 (16%)	2.58	(1.31, 5.05)	0.006
Location left upper	16 (18%)	25 (32%)	0.45	(0.24, 0.85)	0.013
Location left lower	25 (28%)	19 (25%)	1.17	(0.64, 2.14)	0.60
Vein part of common vein	19 (21%)	9 (12%)	2.02	(0.79, 5.20)	0.14

^1^ Values are shown as median (range) or number (%). PVS = pulmonary vein stenosis.

**Table 5:** Vein status at study endpoint, by patient, and vein characteristics: multivariable analysis for the outcome of minimal disease.

	Odds Ratio	95% Confidence Interval	*p* Value
No disease at baseline	6.15	(1.69, 22.4)	0.006
No distal disease at baseline	3.69	(1.52, 8.94)	0.004
Location not left upper	2.58	(1.07, 6.19)	0.034
Age at drug start date			
→ <6 months	1.00	-	-
→ 6 months to <1 year	1.59	(0.63, 4.01)	0.33
→ ≥1 year	5.59	(1.81, 17.3)	0.003

**Table 6:** Rate of reintervention during the study period: univariate analysis.

	Number of Reinterventions ^1^			IRR ^2^	95% Confidence Interval	*p* Value
	None (*n* = 85)	1 (*n* = 25)	≥2 (*n* = 57)			
Patient Level						
Age at drug start date (months)	9 (2, 61)	6 (3, 45)	6 (1, 45)	0.96	(0.89, 1.03)	0.24
Age at drug start date						
→ <6 months	19 (22%)	12 (48%)	30 (53%)	1.00	-	-
→ 6 months to <1 year	45 (53%)	5 (20%)	21 (37%)	0.40	(0.20, 0.81)	0.011
→ ≥1 year	21 (25%)	8 (32%)	6 (11%)	0.29	(0.12, 0.71)	0.007
Sex female	44 (52%)	8 (32%)	25 (44%)	0.87	(0.44, 1.74)	0.70
Premature birth	33 (39%)	9 (36%)	25 (44%)	1.48	(0.73, 3.00)	0.27
Diagnosis primary PVS	4 (5%)	9 (36%)	3 (5%)	0.94	(0.47, 1.88)	0.86
Diagnosis anomalous vein	23 (27%)	8 (32%)	20 (35%)	1.11	(0.54, 2.32)	0.77
Single ventricle physiology	13 (15%)	3 (12%)	7 (12%)	0.73	(0.29, 1.81)	0.49
Presence of lung disease	28 (33%)	7 (28%)	24 (42%)	1.50	(0.75, 2.99)	0.25
Genetic syndrome	16 (19%)	8 (32%)	21 (37%)	1.13	(0.59, 2.17)	0.72
Percent of eligible drug doses received (↑5%) (*n* = 79, 23, 57)	0.90 (0.54, 0.99)	0.89 (0.54, 0.98)	0.81 (0.55, 0.92)	0.78	(0.68, 0.89)	<0.001
Surgery type						
→ Conventional sutureless	34 (40%)	14 (56%)	24 (42%)	1.00	-	-
→ Modified sutureless	35 (41%)	11 (44%)	23 (40%)	0.78	(0.36, 1.65)	0.51
→ None/other	16 (19%)	0 (0%)	10 (18%)	0.74	(0.27, 2.00)	0.55
Vein Level						
Distal disease at baseline	12 (14%)	8 (32%)	25 (44%)	1.79	(1.09, 2.96)	0.022
No disease at baseline	24 (28%)	2 (8%)	1 (2%)	0.19	(0.04, 0.90)	0.036
Location right upper	22 (26%)	7 (28%)	12 (21%)	0.75	(0.48, 1.19)	0.22
Location right lower	24 (28%)	7 (28%)	10 (18%)	0.80	(0.57, 1.14)	0.22
Location left upper	17 (20%)	8 (32%)	16 (28%)	1.17	(0.83, 1.66)	0.37
Location left lower	22 (26%)	3 (12%)	19 (33%)	1.31	(0.96, 1.79)	0.090
Vessel part of common vein	23 (27%)	3 (12%)	2 (4%)	0.19	(0.06, 0.63)	0.007

^1^ Values are shown as median (range) or number (%). ^2^ IRR = Incidence Rate Ratio, PVS = pulmonary vein stenosis.

**Table 7:** Rate of reintervention during the study period: multivariable analysis.

	IRR	95% Confidence Interval	*p* Value
No disease at baseline	0.18	(0.05, 0.67)	0.011
Percent of eligible drug doses received (↑5%)	0.76	(0.68, 0.85)	<0.001
Vessel part of common vein	0.17	(0.05, 0.55)	0.003

IRR = Incidence Rate Ratio.

## Data Availability

The data presented in this study are available on request from the corresponding author. The data are not publicly available in order to maintain patient privacy.
